# Cervical Papanicolaou Smear Test Screening among Patients Visiting the Outpatient Department of Gynaecology of a Tertiary Care Centre

**DOI:** 10.31729/jnma.8256

**Published:** 2023-09-30

**Authors:** Ram Prasad Sapkota, Sunilmani Pokhrel, Anita Bhandari, Pradeep Adhikari, Mabindra Shrestha

**Affiliations:** 1 Department of Obstetrics and Gynaecology, Bharatpur Hospital, Bharatpur, Chitwan, Nepal; 2 Department of Pathology, Bharatpur Hospital, Bharatpur, Chitwan, Nepal; 3 Department of Anaesthesia, Bharatpur Hospital, Bharatpur, Chitwan, Nepal

**Keywords:** *cervical cancer*, *cytology*, *papanicolaou smear*

## Abstract

**Introduction:**

Carcinoma cervix is the second most common cause of death in women worldwide and the most common cause in developing countries. Cervical cancer is considered a preventable gynaecological problem as it has a long premalignant stage which can be detected by exfoliative cytology like papanicolaou smear test. The papanicolaou smear test is a simple, safe, non-invasive, and low-cost effective method for screening cervical cancer in developing countries like Nepal. The aim of the study was to find out the prevalence of cervical papanicolaou smear test screening among patients visiting the Outpatient Department of Gynaecology of a tertiary care centre.

**Methods:**

A descriptive cross-sectional study was conducted among patients visiting the Department of Gynaecology of a tertiary care centre after obtaining ethical approval from the Institutional Review Committee. Data from 14 April 2021 to 22 October 2022 were collected between 11 May 2023 to 26 May 2023 from the hospital records. Papanicolaou smear tests among the age group of 21 years up to 70 years were included in the study. Convenience sampling method was used. The point estimate was calculated at a 95% Confidence Interval.

**Results:**

Among 11,173 patients, papanicolaou smear test was done in 572 (5.12%) (4.71-5.53, 95% Confidence Interval). Negative for intraepithelial lesion was the most common cytological pattern seen in 518 (90.55%) patients. The low-grade squamous intraepithelial lesion was the most common among abnormal epithelial cells seen in 29 (5.07%).

**Conclusions:**

The prevalence of cervical papanicolaou smear test among patients visiting the Outpatient Department of Gynaecology was found to be similar to other studies done in similar settings.

## INTRODUCTION

Carcinoma cervix is the second most common cause of death in women worldwide and the most common cause in developing countries.^1^ Within Nepal, cervical cancer continues to be the leading cancer among women, with an annual incidence of 2,244 new cases and 1,493 deaths. Nepal has a cervical cancer incidence of 16.4 per 100,000 women, in contrast to the WHO's desired target of 4 per 100,000 women, nearly four times the target to eliminate the public health issue of cervical cancer.^2^

Papanicolaou (PAP) smear is a simple, safe, noninvasive, outdoor, and low-cost effective screening method for the detection of cervical cancer.^3^ Due to the long pre-invasive state of cervical cancer and earlier identification by screening methods and effective treatment in a non-invasive state, cervical cancer is considered a preventable disease. The goal of cervical cancer screening is to find precancerous cervical cell changes when treatment can prevent cervical cancer from developing.

The aim of the study was to find out the prevalence of cervical papanicolaou smear test screening among patients visiting the Outpatient Department of Gynaecology of a tertiary care centre.

## METHODS

This descriptive cross-sectional study was conducted in the Outpatient Department of Gynaecology of Bharatpur Hospital, Bharatpur, Chitwan, Nepal. Data from 14 April 2021 to 22 October 2022 were collected between 11 May 2023 to 26 May 2023 from the hospital records. Ethical approval for the study was taken from the Institutional Review Committee of the same institute (Reference number: 079/80-020). The age of the patient and PAP smear results were retrieved from the record book in the Department of Pathology. PAP smear tests done among the age group of 21 years up to 70 years were included in the study. PAP reports of samples collected from institutes other than Bharatpur Hospital were excluded from the study. Convenience sampling method was used. The sample size was calculated using the formula:


$$\begin{array}{ll} \text{n} & ={\text{Z}}^{2}\times \frac{\text{p}\times \text{q}}{{\text{e}}^{\text{2}}}\\ & =1.96^{2}\times \frac{0.50\times 0.50}{0.02^{2}}\\ & =2401 \end{array}$$

Where,

n = minimum required sample size
Z = 1.96 at 95% Confidence interval (CI)
p = prevalence taken as 50% for maximum sample size calculation
q = 1-p
e = margin of error, 2%

The minimum required sample size was 2401. However, the final sample size taken was 11,173.

The 2014 Bethesda System of Classification of PAP smear was used for reporting which consists of, Negative for intraepithelial lesion for malignancy (NILM), unsatisfactory for evaluation or inadequate sample, atypical squamous cell of undetermined significance (ASCUS), atypical glandular cell (AGC), low grade squamous intraepithelial lesion (LSIL), high grade squamous intraepithelial lesion (HSIL) and carcinoma or malignant.^5^ Various cytological patterns of PAP smear samples were noted and studied.

Data was entered and analysed by using Microsoft Excel 2016. The point estimate was calculated at a 95% CI.

## RESULTS

Among 11,173 patients, PAP smear was done in 572 (5.12%) (4.71-5.53, 95% CI). The most common age group for the PAP smear test was between 31-40 years, 249 (43.53%) and the least common was among the age group of 61-70 years, 21 (3.67%) (Table 1).

**Table 1:** Age-wise distribution of patients undergoing PAP smear test (n= 572).

Age group (years)	n (%)
21-30	102 (17.83)
31-40	249 (43.53)
41-50	171 (29.89)
51-60	29 (5.06)
61-70	21 (3.67)

NILM is the most common cytological pattern seen in 518 (90.55%) cases. However, AGC and malignancy are the least common which is seen in 1 (0.17%) in both cases.

**Table 2:** Cytological pattern in PAP smear test (n = 572).

Cytodiagnosis		n (%)
NILM	Normal	128 (22.37)
	Non-specific inflammatory	341 (59.61)
	Gardnerella	45 (7.86)
	Trichomonas	4 (0.69)
LSIL		29 (5.07)
**Inadequate**		17 (2.97)
HSIL		4 (0.69)
ASCUS		2 (0.35)
AGC		1 (0.17)
**Malignant**		1 (0.17)

## DISCUSSION

In our study, the prevalence of PAP smear tests among patients visiting the Outpatient Department of Gynaecology was found to be 572 (5.11%) which is similar to the study done at Dhulikhel Hospital (5.9%).^4^ In our study, the maximum number of patients were in the age group of 31 to 40 years, 249 (43.53%) which is similar to the study done at different institutes of Nepal and India where the maximum number of patients in the same age group were 39.04%, 38.3%, and 45.3% respectively.^4-6^

Even though the highest number of patients were in the age group of 31 to 40 years, the prevalence of PAP smear tests was less in studies done at different institutes in India where the prevalence was found to be 33.66% and 33%.^7,8^ This may be due to the wider range of age groups in both the study. We have included 21 to 70 years but in the study done at Queen Mary's Hospital, part of King George Medical University, India included from 20 years up to any age group and at Government Medical College, Punjab, India included from age 11 yrs up to 100 yrs.^7,8^

Conventional PAP test was found to have a false negative rate of about 14-33% approximately two-thirds of which is due to the limitation of sampling and slide preparation.^9^ These limitations may lead to inaccuracy and equivocal diagnosis. In our study, 2.97% of the PAP smear samples were inadequate which is similar to the study done at Punjab, India (4%) but less than the study done at Karnataka, India (9.3%). The higher incidence of unsatisfactory samples may be due to the involvement of a higher age group of up to 100 years and the non-accessibility of squamocolumnar junction in cases of post-menopausal women.

The prevalence of NILM is 90.55% in our study which is similar to the study done at different institutes of India and Bangladesh where the prevalence was found to be 91%,^6^ 91.5%,^7^ and 91.8%.^10^ However, it is lesser in the study done at tertiary care hospital, India (82.08%).^8^ In our study, the prevalence of abnormal epithelial cells is 6.46% which is similar to the study done in Saudi Arabia, 5% and Nepal, 6.54%.^5,11^ In our study, LSIL is the commonest 29 (5.07%) epithelial cell abnormality which is similar to the study done in Bangladesh, 6.36% but lesser in studies done in Nepal,1.6% and India, 2.7%.^5,6,10^ In our study the prevalence of ASCUS was 0.35% which is consistent with the study done at Bangladesh, 0.18% and India, 0.3% but it is lesser than the study done in Nepal which was 3.6%.^5,6,10^ The prevalence of AGC is 1 (0.17%) in our study which is similar to the study done in Bangladesh (0.12%).^10^ In our study, the prevalence of HSIL is 4 (0.69%) which is consistent with the study done at Nepal and different institutes of India where it was found to be 0.9%, 0.64%, and 1.18% respectively.^5,8,10^ In similar studies done in Nepal and Bangladesh malignant cells were seen in 0.26% and 0.35% which were similar to our study where it was found to be 1 (0.17%) whereas it was lower than another study done in India (1.3%).^4,6,10^

This study has few limitations. Since our study was single centred study and a small sample size was taken within a limited time frame, it cannot be generalised to the general population. Furthermore, since this is a descriptive study, we were unable to find associations between the variables and cytological patterns of epithelial cells in the PAP Smear Test.

## CONCLUSIONS

The prevalence of cervical papanicolaou smear test among patients visiting the Outpatient Department of Gynaecology was found to be similar to other studies done in similar settings.
